# Identification of ACE and HSPB8 as novel drug targets for LUSC treatment and prognosis based on a prognostic model integrating epigenetic regulation and endoplasmic reticulum stress-related genes

**DOI:** 10.1371/journal.pone.0335395

**Published:** 2026-01-05

**Authors:** Yicong Zhou, Bin Wang, Changliang Song, Wenyao Xie

**Affiliations:** 1 Department of Neurology, Handan Central Hospital, Hebei, Handan, P.R. China; 2 Department of Oncology, Handan Central Hospital, Hebei, Handan, P.R. China; Shantou University Medical College, CHINA

## Abstract

**Background:**

Lung Squamous Cell Carcinoma (LUSC) remains a significant challenge in oncology due to limited diagnostic and therapeutic options. Epigenetic regulation and endoplasmic reticulum stress (ERS) play crucial roles in cancer pathogenesis, progression, and immune evasion, making them valuable areas of investigation for understanding LUSC.

**Methods:**

This study integrated data from The Cancer Genome Atlas (TCGA) and the Genotype-Tissue Expression (GTEx) project to identify differentially expressed epigenetics and endoplasmic reticulum stress-related genes (EERSGs) in LUSC. Using the CIBERSORT algorithm, tumor-infiltrating immune cells were analyzed, and various machine learning and Cox models were employed to filter characteristic and prognostic markers. Further investigations included single-gene analysis, pan-cancer exploration, validation using the Human Protein Atlas (HPA) database, drug sensitivity analysis, and experimental validation through knockdown models, Western Blot, CCK-8, and Transwell experiments.

**Results:**

Our analysis identified ACE and HSPB8 as potential therapeutic targets and prognostic markers in LUSC. A risk scoring system was developed, accurately predicting patient survival outcomes. Low expression groups were associated with decreased immune escape potential, indicating higher efficacy of chemotherapy and immunotherapy. Quantitative analysis of tumor-infiltrating immune cells (TIICs) and functional assays revealed significant differences in the roles of ACE and HSPB8 between normal lung and LUSC cells, underscoring their importance in tumor biology and epigenetic regulation.

**Conclusion:**

This comprehensive bioinformatics and experimental study highlights ACE and HSPB8 as novel targets for LUSC treatment and prognosis, emphasizing their roles in epigenetic regulation and ERS. The risk model demonstrates preliminary potential for guiding personalized therapeutic strategies, emphasizing the need for a deeper understanding of epigenetic mechanisms and ERS in cancer development and treatment. Thus, our findings open avenues for further research into targeted therapies for LUSC, aiming to improve patient outcomes through precision medicine that considers both epigenetic factors and ERS.

## 1. Introduction

Lung cancer remains the leading cause of cancer-related mortality worldwide. It is primarily categorized into two main types: small cell lung cancer and non-small cell lung cancer (NSCLC), with NSCLC accounting for approximately 85% of all lung cancer cases. NSCLC predominantly comprises lung adenocarcinoma (LUAD) and lung squamous cell carcinoma (LUSC), with the latter constituting about 30% of all NSCLC cases [1–3]. As a subtype of NSCLC characterized by a particularly poor prognosis, LUSC presents two significant challenges: first, the early diagnosis of LUSC is exceedingly difficult [4]; second, compared to other tumor types, therapeutic options for LUSC are relatively limited [5]. Notably, due to the lower frequency of EGFR mutations, ALK rearrangements, or ROS1 fusions in LUSC [6,7], there is a poor response to currently available targeted therapies. Consequently, the identification of LUSC-related biomarkers and the exploration of novel targeted treatment strategies have become focal points in current clinical research [8–10].

Proteins, as the executors of biological functions, undergo synthesis, folding, and modification primarily within the endoplasmic reticulum (ER), a process that is strictly regulated and directly impacts cellular function, fate, and viability [11]. However, these processes are influenced not only by gene sequences but also significantly by epigenetic regulation. Epigenetic modifications, such as DNA methylation and histone modifications, can modulate gene expression, thereby affecting protein production and function [12]. Various external stimuli and internal events may alter the epigenetic landscape, influencing the ER’s protein folding capacity, potentially leading to the accumulation of misfolded or unfolded proteins. This accumulation can trigger endoplasmic reticulum stress (ERS) and activate the unfolded protein response (UPR) [13]. Through the UPR, ERS can induce various cell death pathways via three primary transmembrane protein receptors on the ER membrane: inositol-requiring enzyme 1α (IRE1α), PKR-like ER kinase (PERK), and activating transcription factor 6 (ATF6) [14]. In tumor biology, alterations in the tumor microenvironment (TME) have been found to trigger ERS in both malignant cells and infiltrating immune cell populations [15–17]. The current state of ERS has been shown to regulate the dynamic reprogramming of various precancerous characteristics and cellular functions, indicating a crucial role of ER stress sensors and their downstream signaling pathways in modulating tumor growth, metastasis, and responses to chemotherapy, targeted therapy, and immunotherapy [15,18]. Studies have also demonstrated that interventions targeting ER stress signaling can directly eliminate lung squamous carcinoma cells and elicit anti-cancer immune responses [16,19].

The objective of this study is to integrate LUSC-related datasets to identify differentially expressed genes (DEGs) and perform an integrated analysis with epigenetics and endoplasmic reticulum stress-related genes (EERSGs). Utilizing CIBERSORT and machine learning algorithms, this research aims to explore immune patterns and biological networks, and, in conjunction with overall survival (OS) follow-up data, to construct risk models. Based on these analyses, a series of experiments simulating real cellular gene expression mechanisms were designed to further investigate genetic variations in LUSC and screen for potential biomarkers related to epigenetics and ER stress. The ultimate goal is to provide novel therapeutic and prognostic targets for LUSC and, more broadly, for cancer treatment.

## 2. Materials and methods

### 2.1 Data source

The data used in this study were sourced from three main databases. Firstly, we obtained a dataset from The Cancer Genome Atlas (TCGA) database (https://portal.gdc.cancer.gov/) that includes data on 501 LUSC samples and 49 normal samples, of which 500 LUSC samples had available survival information [20]. Secondly, 578 normal samples were acquired from the Genotype-Tissue Expression (GTEx) database (https://www.genome.gov/Funded-Programs-Projects/Genotype-Tissue-Expression-Project) [21]. Lastly, epigenetics and endoplasmic reticulum stress-related genes were searched in the GeneCards database (https://www.genecards.org/), retaining genes with relevance scores above 5 to construct the EERSGs [22].

During the data processing phase, data in the format of transcripts per million (TPM) were extracted from STAR-counts data along with clinical information. The data were then normalized using log2 (TPM + 1) transformation to stabilize variance and improve the normal distribution of the data. Finally, only samples with both RNA sequencing data and complete clinical information were retained for subsequent analysis.

### 2.2 Identification of differentially expressed EERSGs

To identify genes with significant expression differences between normal samples and primary LUSC samples, this study utilized the limma and DESeq2 packages to assess gene expression differences in the dataset. The selection of DEGs was based on the criteria of an absolute log fold change (|LogFC|) greater than 2 and an adjusted p-value less than 0.05 after Benjamini-Hochberg correction. This step aims to ensure that the identified genes are statistically significant and that their expression differences have biological relevance. Subsequently, EERSGs were extracted from the GeneCards database.

### 2.3 Protein-protein interaction and enrichment analysis

To further explore the interactions among differentially expressed EERSGs, this study utilized the online platform of the STRING database (https://string-db.org/) to construct a protein-protein interaction (PPI) network [23]. This step aims to reveal the potential interactions and functional connections among differentially expressed EERSGs, providing a foundation for understanding their roles in the development of LUSC. Moreover, to gain deeper insights into the molecular functions of target genes, this study conducted functional enrichment analysis using Gene Ontology (GO) and the Kyoto Encyclopedia of Genes and Genomes (KEGG). The GO analysis offered comprehensive annotations of the molecular functions (MF), biological processes (BP), and cellular components (CC) of differentially expressed EERSGs, revealing their functional localization within the cell and the biological processes they are involved in [24]. The KEGG analysis provided insights into the biological pathways and disease processes involving differentially expressed EERSGs, helping to identify potential intervention targets in the development of lung squamous cell carcinoma [25]. The ClusterProfiler package in R (version: 3.18.0) was used to annotate the enrichment results of GO and KEGG [26].

### 2.4 Analysis of immune infiltrating cells in LUSC

The CIBERSORT algorithm is an advanced computational method designed to accurately estimate the composition of cell types within complex tissues from gene expression profile data. This algorithm is based on the principles of linear support vector regression and utilizes a predefined reference gene expression signature matrix, known as LM22, to perform deconvolution analysis of RNA sequencing data. The LM22 signature matrix includes characteristic expression profiles for 22 immune cell types, enabling CIBERSORT to estimate the relative abundance of these cell types at the RNA transcript level. This allows for the identification and quantification of various cell types within tissue samples.

In this study, we applied the CIBERSORT algorithm to analyze the distribution of 22 immune cell types in LUSC and its corresponding normal tissues. This analysis aims to reveal the differences in immune cell distribution between two types of immune response tissues, thereby exploring changes in the immune microenvironment [27]. Through further computation, we also investigated the functional relationships between these immune cells [28,29]. The results are presented using box plots, bar graphs, and other visualizations.

### 2.5 Signature screening with machine learning methods

To accurately assess the significance of differentially expressed EERSGs, this study employed a range of advanced machine learning algorithms. Initially, the dataset was divided into 60% for the training set and 40% for the test set, with the use of ten-fold cross-validation to ensure the robustness and reliability of the results. The models selected for this study include logistic regression, support vector machine (SVM), naive Bayes, K-nearest neighbors (KNN), neural networks, decision trees, random forests, eXtreme Gradient Boosting (XGBoost), and Light Gradient Boosting Machine (LightGBM).

Logistic regression uses the Sigmoid function to map features to probabilities for classification [30]. Support Vector Machine (SVM) constructs an optimal hyperplane to maximize class separation [31]. Naive Bayes, based on Bayes’ theorem, assumes feature independence for probabilistic classification [32]. K-Nearest Neighbors (KNN) classifies based on the distance between feature points [33]. Neural networks simulate brain functions, learning complex patterns by adjusting network weights and biases [34]. Decision trees classify data through incremental feature-based branching [35]. Random forests ensemble multiple decision trees to enhance accuracy [36]. XGBoost sequentially adds tree models and optimizes the loss function for improved prediction [37]. LightGBM, building on XGBoost’s approach, is optimized for large-scale data [38].

High-weight features identified from multiple top-performing machine learning algorithms were intersected to construct a multi-gene prognostic model. We performed randomized hyperparameter search with 5-fold stratified cross-validation on the training set (shuffle = TRUE, random_state = 42), optimizing ROC-AUC for imbalanced data. To prevent data leakage, all preprocessing steps (e.g., scaling, imputation, feature selection) were included in a single pipeline and fitted within each training fold only. The model’s predictive accuracy and generalizability were evaluated using the concordance index (C-index) and area under the receiver operating characteristic curve (AUC).

### 2.6 Identification of immune subtypes

To understand the heterogeneity in immune response patterns within LUSC, this study employed consensus clustering to analyze immune subtypes based on key gene expression patterns. The ‘ConsensusClusterPlus’ R package [39] was used to ensure analysis consistency and reliability. The clustering was configured with a maximum of 6 clusters, 100 iterations, and 80% random sampling per iteration. Hierarchical clustering with ward. D2 linkage method was applied to optimize cluster cohesiveness.

The study then visualized immune subtype classifications through survival curves, assessing their connection to patient prognosis. Gene expression heatmaps were generated using the ‘pheatmap’ R package, incorporating only genes with a variance greater than 0.1 to ensure result significance and interpretability.

### 2.7 Risk model construction and validation

Lasso method identified genes significantly associated with prognosis. The coefficients of the selected features are determined by the λ parameter. And the multivariate Cox regression model was then constructed to calculate a risk score for each patient using the formula:


$$Risk\;Score=\sum_{i\;=1}^{n}\left(Coefi\times Expi\right)\mathrm{\backslash ]}$$

Where ‘n’ is the number of feature genes, ‘Coefi’ is the λ coefficient, and ‘Expi’ is the gene expression level.

Patients were divided into high-risk and low-risk groups based on the median risk score. The ‘survival’ package was used to plot risk curves, and Kaplan-Meier (KM) method compared survival differences between groups. ROC curves and log-rank tests evaluated the correlation between the risk model and clinical characteristics.

The study incorporated the AJCC staging system [40] and the chromosomal instability feature (CIN25) [41,42] to validate the Cox regression model results. This approach enhances the accuracy and reliability of LUSC prognosis assessment on both statistical and biological levels.

### 2.8 Gene characteristics analysis

This study conducted an in-depth analysis of the selected target genes and their relationships with immune cells, tumor mutation burden (TMB), and stemness scores. The ‘ggstatsplot’ package was used to visualize Spearman correlation analyses.

TMB, an important quantitative biomarker, is defined as the total number of somatic mutations per megabase of the tumor genome, excluding germline mutations. High TMB levels in tumor cells can enhance the recognition of new antigens by the immune system, facilitating the activation of anti-tumor T-cell proliferation and response [43].

The stemness score, measuring the similarity between tumor cells and stem cells, is quantified through mRNA expression instability (mRNAsi). This is calculated using the OCLR (Oncogenicity-Correlated LncRNA Resilience) algorithm developed by Malta et al. [44], based on a comprehensive gene expression profile analysis involving 11,774 genes. For intuitive representation, the stemness scores were linearly transformed to a standardized scale of 0–1, by subtracting the minimum value and dividing by the maximum value.

### 2.9 Pan-cancer exploration

To explore a gene’s role across pan-cancers, we employed both visual and statistical analyses. Box plots were used to visually represent the gene’s expression levels across various tumor types, providing an intuitive comparison across different cancers. To assess the gene’s prognostic value, we conducted univariate Cox regression analysis, evaluating the association between gene expression levels and patient survival outcomes. The results were visualized using forest plots, constructed with the ‘forestplot’ package, displaying hazard ratios (HR), 95% confidence intervals (95% CI), and corresponding P-values.

This approach, combining expression level visualization and prognostic analysis, allows for a comprehensive examination of the gene’s expression patterns and their potential impact on patient prognosis across various cancer types. It provides valuable insights into the gene’s pan-cancer role, contributing to a deeper understanding of its significance in different malignancies.

### 2.10 HPA pathology validation

We validated the protein expression of relevant genes in LUSC using the Human Protein Atlas (HPA) database. The HPA (https://www.proteinatlas.org/) is a comprehensive and authoritative resource for mapping the spatial distribution of proteins in human tissues and cells [45]. By integrating cutting-edge transcriptomics and proteomics technologies, the HPA offers detailed information on protein expression across various human tissues and organs at both RNA and protein levels. This approach enables a thorough validation of protein expression for genes of interest in LUSC, providing valuable insights into their biological significance and potential therapeutic implications.

### 2.11 Chemotherapy drug analysis

The Genomics of Drug Sensitivity in Cancer (GDSC) database (https://www.cancerrxgene.org/) is a key resource for understanding the relationship between genomic variations and drug responses in cancer [46]. We utilized this database to predict drug sensitivity in LUSC samples.

Using the ‘pRRophetic’ package, we constructed ridge regression models based on cell line and LUSC gene expression profiles. This allowed us to estimate the half-maximal inhibitory concentration (IC50) for various anticancer drugs [47], comparing sensitivity between high-risk and low-risk groups.

Our methodology accounted for batch effects and tissue types, using the combat method for correction and setting all tissue types to “all” for broad applicability. We averaged repeated gene expression data to enhance reliability. All parameters were kept at default to ensure reproducibility.

### 2.12 SiRNA formulations

In this study, we utilized the normal lung cell line BEAS-2B and the LUSC cell line H520, both provided by the Chinese Academy of Sciences. For the feature genes, we selected corresponding target sequences from the NCBI Nucleotide database, specifically from Homo sapiens chromosome 17 and chromosome 12 (GRCh38.p14 Primary Assembly). Small interfering RNA (siRNA) sequences were designed using the Invitrogen Block-iT RNAi Designer tool. The target sequences were: ACE - ‘CAGAATGGCTTTCTGAGCATTGATT’ and HSPB8 - ‘AGTTCCGAAACTGGCTCCAACTTTA’. Cy5-labeled siRNA was synthesized and supplied by RiboBio Company (Guangzhou, PR China).

### 2.13 Western Blot (WB) analysis

To investigate the expression differences of ACE and HSPB8 proteins in BEAS-2B and H520 cell lines, and to evaluate the efficacy of siRNA constructs, we employed Western blot analysis. Total protein was extracted using RIPA lysis buffer (Beyotime, Shanghai, China). Proteins were separated by sodium dodecyl sulfate-polyacrylamide gel electrophoresis (SDS-PAGE) and transferred to PVDF membranes (MilliporeSigma, Germany) using a cold transfer buffer and semi-dry transfer system.

Membranes were blocked in 5% skim milk for 1 hour, then incubated overnight at 4°C with primary antibodies targeting ACE, HSPB8, and glyceraldehyde-3-phosphate dehydrogenase (GAPDH) (Proteintech, Wuhan, China) [48]. The following day, membranes were incubated with corresponding HRP-conjugated secondary antibodies for 1 hour. Signal detection was performed using enhanced chemiluminescence (ECL) reagent (MilliporeSigma, Germany) according to the manufacturer’s instructions.

Protein band density was quantified using ImageJ software. GAPDH served as an internal reference protein for normalization to calculate the relative expression levels of the target proteins.

### 2.14 CCK-8 assay

To assess the cell proliferation capacity, six groups were seeded into 96-well plates: normal cell line + blank siRNA, LUSC cell line + blank siRNA, normal cell line + ACE-siRNA, LUSC cell line + ACE-siRNA, normal cell line + HSPB8-siRNA, and LUSC cell line + HSPB8-siRNA. Following the manufacturer’s instructions, 10 µL of CCK-8 solution (Beyotime, Shanghai, China) was added to each well. The absorbance (OD value) was measured at a wavelength of 450 nm using a microplate reader. The measurements were recorded every 24 hours for 3 days to evaluate the cell proliferation capability.

### 2.15 Transwell assay

For the migration assay, cell suspensions were adjusted to a proper density, typically 1 × 10^5 cells/mL. In the upper chamber of the Transwell (Corning, New York, USA), 200 µL of cell suspension (in serum-free medium) was added, and 600 µL of complete medium containing 10% FBS was added to the lower chamber as a chemoattractant. The Transwells were then incubated at 37°C and 5% CO2 for 48 hours to allow for cell migration. After the migration period, the Transwells were washed with PBS to remove non-adherent cells, then cells were fixed with 4% formaldehyde for 20 minutes, and stained with crystal violet (Beyotime, Shanghai, China) for 20 minutes. A cotton swab was used to gently remove non-migrated cells from the upper chamber, and the cells that had migrated through the membrane to the lower chamber were counted under an inverted microscope.

For the invasion assay, the procedure builds on the migration assay with the addition of Matrigel (Corning, New York, USA) diluted with serum-free medium at a 1:3 ratio or as required by the experiment. 50 µL of this mixture was added to the upper chamber of the Transwell and incubated at 37°C for 1 hour to allow the Matrigel to solidify, creating a barrier that mimics the extracellular matrix and requires cells to invade through this layer to migrate.

All CCK-8 assays, Transwell assays, and Western blotting were performed in at least three independent biological replicates with two technical replicates each. Statistical comparisons between two groups used Student’s t-test or Wilcoxon rank-sum test, while multi-group comparisons were analyzed using one-way ANOVA followed by Tukey’s post-hoc test. Significance was defined as p < 0.05.

### 2.16 Statistical analysis

All analyses mentioned above were performed using R language (version 4.1.2). The following package versions were used to ensure reproducibility: limma (3.60.2), DESeq2 (1.44.0), clusterProfiler (4.12.0), ConsensusClusterPlus (1.66.0), ggstatsplot (1.4.0), pheatmap (1.0.12), survival (3.5.8), forestplot (3.1.3), pRRophetic (0.5), and the CIBERSORT algorithm within the IOBR package (0.99.0). Differences between two groups were analyzed using the Wilcoxon rank-sum test or Student’s t-test. The correlation analysis among variables was conducted using the Spearman correlation test. All calculated statistical p-values were two-sided, and values of p ≤ 0.05 were considered to be statistically significant, with the following notation for significance levels: ns (not significant): p > 0.05, *: p ≤ 0.05, **: p ≤ 0.01, ***: p ≤ 0.001.

## 3. Results

**Fig 1:**
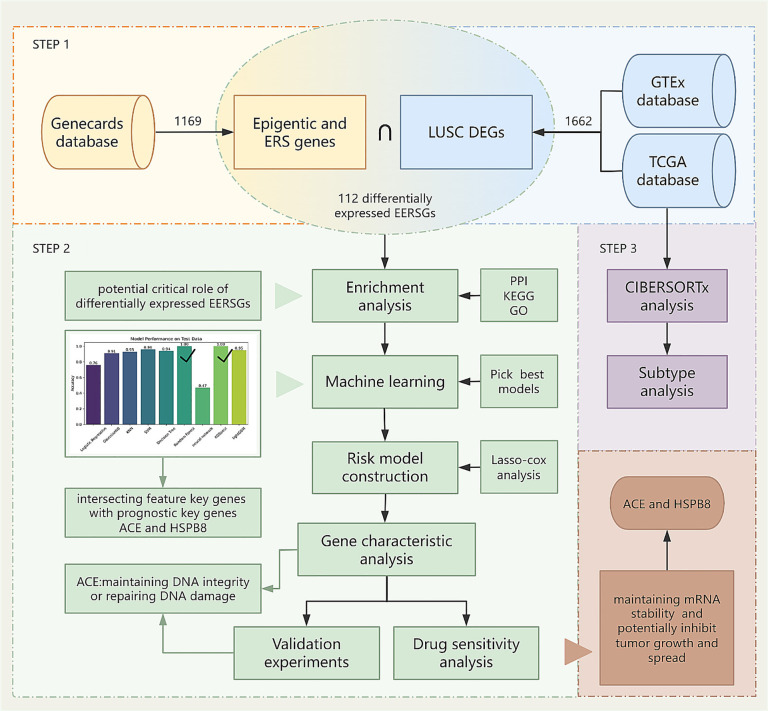
Study flowchart.

All analytical processes are illustrated in the flowchart (Fig 1).

### 3.1 Identification of differentially expressed EERSGs

**Fig 2:**
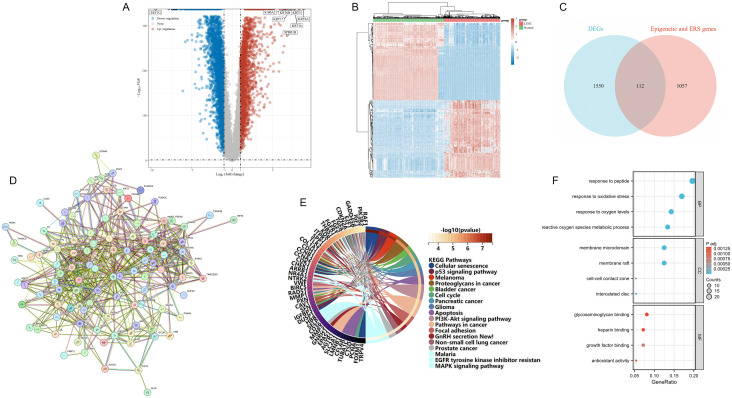
Overview of differential gene expression and enrichment analysis in LUSC. **(A)** Volcano Plot of 1,662 DEGs **(B)** Heatmap of Top 100 Significant DEGs **(C)** Intersection Analysis via Venn Diagram **(D)** PPI Network of 112 differentially expressed EERSGs. **(E)** KEGG Pathway Enrichment: Lines indicate associations between specific pathways and genes. **(F)** GO Analysis Results with three aspects.

Through differential expression analysis between normal and LUSC samples, we identified 1,662 DEGs, comprising 567 upregulated and 1,095 downregulated genes. A volcano plot illustrates the magnitude of change and overall distribution of these DEGs (Fig 2A). Additionally, a heatmap presents the expression of the top 100 most significant DEGs between normal individuals and LUSC patients (Fig 2B). Further intersection analysis between 1,169 EERSGs and the 1,662 DEG set yielded 112 differentially expressed EERSGs for downstream analysis (Fig 2C).

### 3.2 PPI network construction and enrichment analysis

Fig 2D illustrates the protein-protein interaction (PPI) network composed of 112 differentially expressed EERSGs. In-depth analysis of these genes revealed enrichment in several KEGG pathways, including Cellular Senescence, Pathways in Cancer, Non-small Cell Lung Cancer, EGFR Tyrosine Kinase Inhibitor Resistance, and the MAPK signaling pathway. These results highlight the potential critical role of differentially expressed EERSGs in cell proliferation, death, differentiation, cancer development, and treatment resistance (Fig 2E).

GO analysis further revealed functional enrichment in three aspects:

1. BP: Genes were significantly enriched in responses to peptide, oxidative stress, oxygen levels, and reactive oxygen species metabolic processes, revealing their key functions in regulating oxidative stress and reactive oxygen species metabolism.
2. CC: Enrichment results show gene expression related to membrane microdomains, membrane rafts, cell-cell contact zones, and striated muscle contraction zones. These are key sites for intracellular signaling and material exchange, emphasizing the importance of the endoplasmic reticulum in cell function.
3. MF: Analysis revealed roles in glycosaminoglycan binding, heparin binding, growth factor binding, and antioxidant activity. This indicates potential key roles in regulating interactions with the extracellular matrix, promoting cell growth and differentiation, and protecting cells from oxidative damage (Fig 2F).

### 3.3 Feature analysis of immune infiltrating cells in LUSC

**Fig 3:**
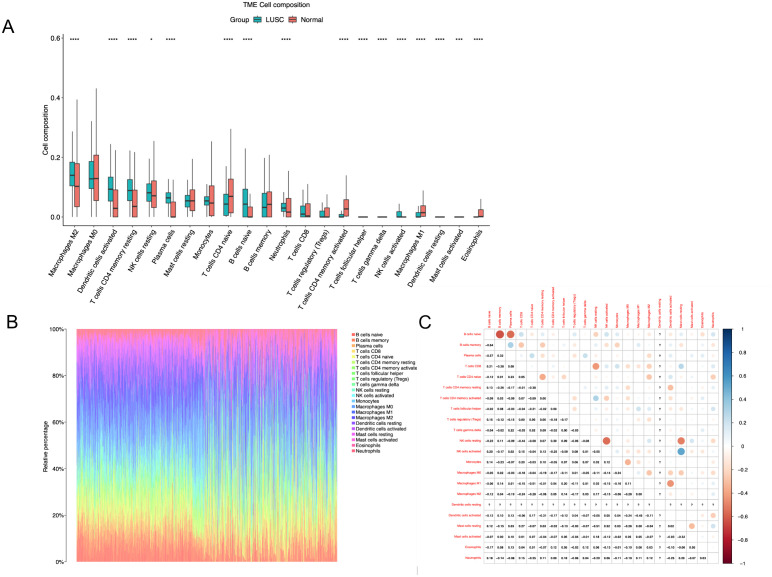
Analysis of immune cell infiltration characteristics in LUSC. **(A)** Box plots depict the distribution of 22 types of immune cells between LUSC and normal tissues. **(B)** Bar plots show the distribution of 22 types of immune cells in each sample, with LUSC samples at the top and normal samples at the bottom. **(C)** Heatmap illustrates the correlation relationships between different cell types in LUSC.

Fig 3A illustrates the distribution of 22 types of immune cells in LUSC and normal lung tissues. Our analysis revealed significant differences in immune infiltration between LUSC and normal tissues, particularly in M2 macrophages, activated dendritic cells, resting memory CD4 + T cells, plasma cells, peripheral blood CD4 + T cells, neutrophils, activated memory CD4 + T cells, follicular helper T cells, γδ T cells, activated NK cells, M1 macrophages, resting dendritic cells, and eosinophils. These findings underscore the critical roles these immune cells may play within the immune microenvironment of LUSC.

Fig 3B presents barplots demonstrating the distribution of these 22 types of immune cells in each sample, highlighting individual variations in immune cell infiltration among LUSC samples.

Fig 3C displays a correlation heatmap exploring the interactions between different types of immune cells in LUSC. The analysis reveals a significant positive correlation between γδ T cells and activated NK cells, while showing significant negative correlations between γδ T cells and resting NK cells, T cells CD4 naive and M2 macrophages, and M0 macrophages and activated dendritic cells.

### 3.4 Signature screening with machine learning methods

**Fig 4:**
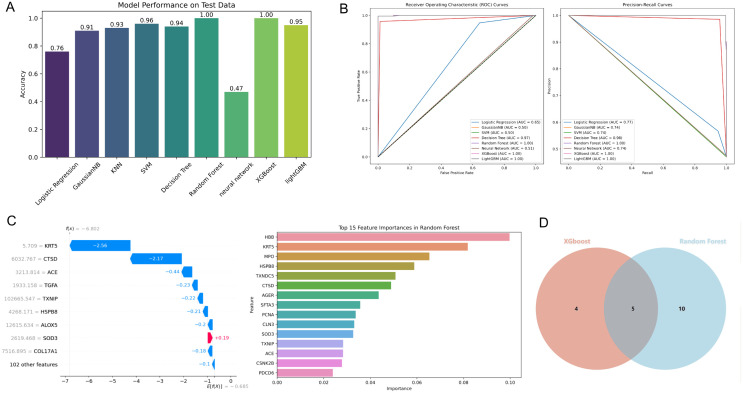
Analysis of machine learning model results. **(A)** Accuracy of Nine Machine Learning Models. **(B)** Evaluation of AUC-ROC and PR Scores. **(C)** High-Weight Genes Identified by Random Forest and XGBoost. **(D)** Venn Diagram for Gene Intersection.

We employed nine different machine learning models for predictive analysis on 112 differentially expressed EERSGs and ranked them based on model accuracy (Fig 4A). To comprehensively evaluate the predictive performance of these models, we utilized the area under the receiver operating characteristic curve (AUC-ROC) and precision-recall (PR) scores for an integrated assessment (Fig 4B). Based on the combined results of AUC-ROC and PR scores, XGBoost and Random Forest emerged as the two best-performing models.

In the XGBoost model, high-weight genes include KRT5, CTSD, ACE, TGFA, TXNIP, HSPB8, ALOX5, SOD3, and COL17A1. The Random Forest model identified HBB, KRT5, MPO, HSPB8, TXNDC5, CTSD, AGER, SFTA3, PCNA, CLN3, SOD3, TXNIP, ACE, CSNK2B, and PDCD6 as high-weight genes (Fig 4C). Further analysis revealed that genes frequently appearing in both models include KRT5, CTSD, ACE, HSPB8, and TXNIP, highlighting the potential pivotal role these genes may play in the endoplasmic reticulum stress response in LUSC (Fig 4D).

### 3.5 Identification of immune subtypes

**Fig 5:**
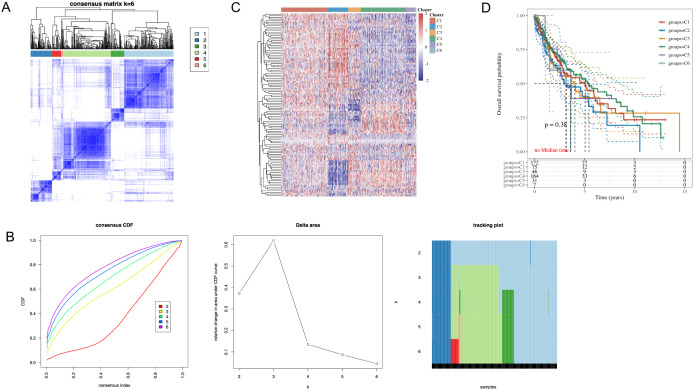
Consensus clustering analysis of differentially expressed EERSGs in LUSC. **(A)** Optimal cluster number (k = 6) selected based on the Cumulative Distribution Function (CDF) Delta area curve, demonstrating the best clustering stability and resolution. **(B)** Comprehensive display of consensus clustering results, including CDF curves, CDF Delta area curve, and tracking plot. **(C)** Heatmap presentation of gene expression patterns of differentially expressed EERSGs across six subgroups. **(D)** KM survival curves for each subgroup.

We performed consensus clustering on the original samples using differentially expressed EERSGs, as illustrated in Fig 5A. The optimal division of samples into six distinct clusters was established, as depicted in Fig 5B. This division was further validated, as demonstrated in Fig 5C, confirming the significant segregation of the samples into six subgroups, each characterized by distinct gene signatures. These findings unveiled the presence of unique gene expression profiles within each subgroup, highlighting the underlying heterogeneity within LUSC.

To explore the clinical implications of our clustering results, we assessed the survival outcomes of the six subgroups using the KM survival analysis method, presented in Fig 5D. Interestingly, no significant statistical differences were observed in the survival curves among the subgroups (p = 0.38). This observation suggests that the heterogeneity identified at the molecular level, while significant, may not directly translate into differential survival outcomes among the subgroups.

It is plausible that the high intra-tumor variability inherent to LUSC, which often leads to the formation of patient-specific clusters, contributes to the observed phenomenon. This variability may mask the potential survival differences across subgroups, underscoring the complex nature of LUSC and the challenge of linking molecular heterogeneity to clinical outcomes directly.

### 3.6 Risk model construction and validation

**Fig 6:**
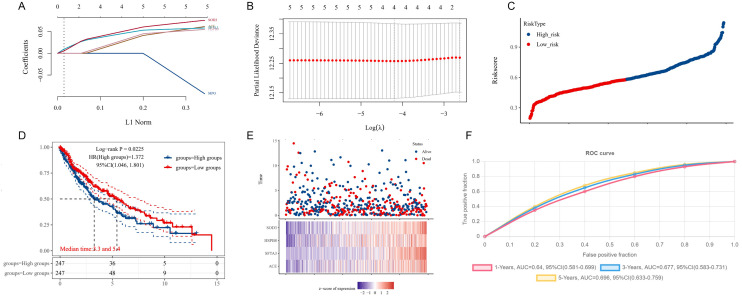
Risk score analysis and survival prediction in LUSC. **(A)** Risk profile in the training set. **(B)** KM survival curves for high-risk and low-risk groups in the training set. **(C)** Distribution of Risk scores for each sample. **(D)** ROC curves for the training set.


Lasso-cox analysis was conducted on high-weight genes from two machine learning models, identifying four genes related to poor survival prognosis: ACE, SFTA3, HSPB8, and SOD3 (Fig 6C). These genes are involved in genetic alterations and regulate tumor immunity, suggesting a potential link to diagnostic efficacy in LUSC cases. Using the expression levels of model genes and regression coefficients obtained from Lasso and Cox regression, the coefficients process is shown in Fig 6A and 6B, the following risk score formula was derived:

Riskscore=(0.0356)*ACE+(0.0504)*SFTA3+(0.0394)*HSPB8+(0.0568)*SOD3\]

Based on the calculated risk scores, samples were categorized into high-risk and low-risk groups (Fig 6C). Kaplan-Meier survival analysis showed significant survival differences between the two groups (Fig 6D), validating the model’s effectiveness. Additionally, we used the AUC to further assess the model’s 1, 3, and 5-year survival predictions, as shown in Fig 6F.

For the final results, we calculated the C-index of the risk score, AJCC staging, and CIN25, with results displayed in Table 1. In the test set, the risk score’s C-index was 0.621, significantly higher than that of the AJCC stage (0.556, p < 0.05) and CIN25 (0.564, p < 0.05). In the training set, the risk score’s C-index was 0.619, significantly higher than the AJCC stage (0.533, p < 0.05) and CIN25 (0.554, p < 0.05).

**Table 1:** C index of risk score, AJCC stage and CIN25.

Methods	Training Set		Test Set	
	C–index(95%CI)	p	C–index(95%CI)	p
Risk score	0.659(0.581,0.737)	–	0.621(0.562,0.680)	–
AJCC stage	0.533(0.467,0.599)	<0.05	0.556(0.479,0.633)	<0.05
CIN25	0.554(0.502,0.606)	<0.05	0.564(0.507,0.621)	<0.05

These results indicate that, compared to other prognostic methods, the risk score can effectively predict patient prognosis. This suggests that our risk score model can efficiently differentiate patient groups with varying survival prognoses in LUSC cases, potentially offering a valuable tool for clinical decision-making and personalized treatment strategies.

### 3.7 Gene characteristics analysis

**Fig 7:**
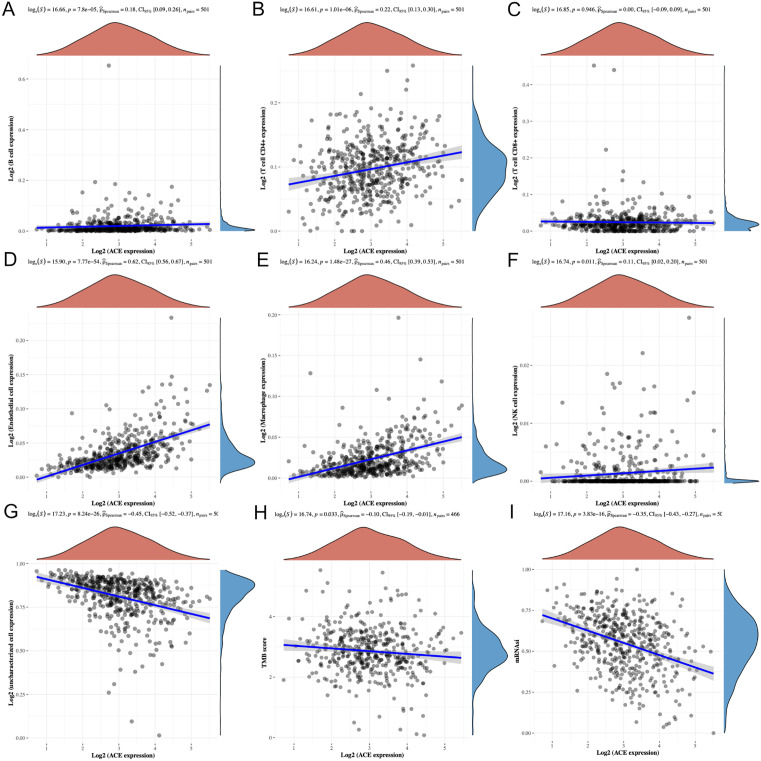
Exploration of ACE characteristics. **(A-G)** Spearman correlation analysis between immune cell types and ACE expression. **(H)** Spearman correlation analysis between TMB and ACE expression. **(I)** Spearman correlation analysis between mRNAsi and ACE expression.

**Fig 8:**
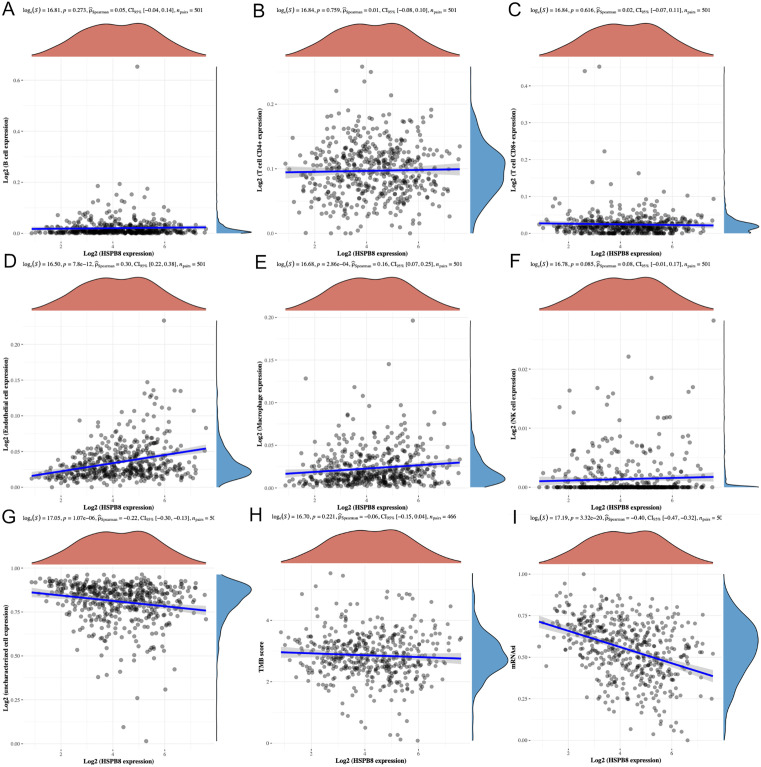
Exploration of HSPB8 characteristics. **(A-G)** Spearman correlation analysis between immune cell types and HSPB8 expression. **(H)** Spearman correlation analysis between TMB and HSPB8 expression. **(I)** Spearman correlation analysis between mRNAsi and HSPB8 expression.

By intersecting feature key genes from machine learning models with prognostic key genes, ACE and HSPB8 were identified for in-depth subsequent analysis. Initially, ACE exhibited significant Spearman positive correlations with B cells, CD4 + T cells, endothelial cells, macrophages, and NK cells (Fig 7A–7G), while HSPB8 showed Spearman positive correlations with endothelial cells and macrophages (Fig 8A–8G). Additionally, both displayed negative correlations with uncharacterized cells. These results suggest that high expression of ACE and HSPB8 may be associated with better responsiveness in immune therapy reactions.

Further analysis revealed a negative correlation between the ACE gene and tumor mutational burden (TMB) (Spearman correlation coefficient of −0.10, P-value of 0.033) (Fig 7H), potentially reflecting ACE’s role in maintaining DNA integrity or repairing DNA damage, thereby affecting the accumulation of mutations. In contrast, no significant correlation was observed between HSPB8 and TMB (Fig 8H).

After that, significant negative correlations were found between both ACE and HSPB8 with mRNA stemness index (mRNAsi) (Spearman correlation coefficient of −0.35, P-value of 3.83e-16 for ACE, and −0.40, P-value of 3.32e-20 for HSPB8) (Figs 7I and 8I). The negative correlation implies that an increase in gene expression levels is associated with a decrease in mRNAsi, and vice versa. As mRNAsi serves as an indicator reflecting tumor heterogeneity and complexity, its reduction may be related to increased differentiation of tumor cells, slowed tumor growth, or decreased tumor invasiveness.

These findings indicate that ACE and HSPB8 may play roles in maintaining mRNA stability and potentially inhibit tumor growth and spread. The positive correlations with various immune cell types suggest their potential involvement in enhancing immune responses within the tumor microenvironment. The negative correlation with TMB for ACE implies a possible role in genomic stability, while the negative correlations with mRNAsi for both genes suggest their potential in promoting tumor cell differentiation or reducing tumor aggressiveness.

Collectively, these results highlight ACE and HSPB8 as promising targets for lung squamous cell carcinoma treatment. Their multifaceted roles in immune cell interactions, potential impact on genomic stability, and influence on tumor stemness characteristics make them attractive candidates for further investigation in developing targeted therapies or as prognostic markers in lung squamous cell carcinoma.

### 3.8 Pan-cancer exploration

**Fig 9:**
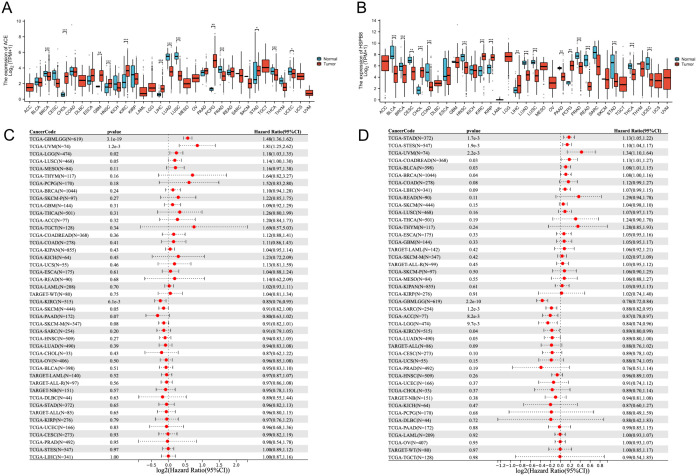
Expression and survival impact of ACE and HSPB8 across cancer types. **(A, B)** Distribution of ACE and HSPB8 expression in various cancer tissues compared to normal tissues, where the horizontal axis represents different cancer types and the vertical axis shows gene expression levels, with different colors indicating different groups. **(C, D)** Results of univariate Cox analysis for the ACE and HSPB8 genes across multiple cancer types.

We constructed a pan-cancer expression profile for the ACE and HSPB8 genes (Fig 9A and 9B). For the ACE gene, we observed significant upregulation in CHOL, KIRC, LIHC, GBM, PCPG, and STAD. Significant downregulation was observed in BRCA, LUAD, LUSC, PRAD, THCA, and UCEC (Fig 9A). The HSPB8 gene showed significant upregulation in CHOL, KIRC, KIRP, LIHC, and PCPG. Significant downregulation was observed in BLCA, BRCA, CESC, COAD, HNSC, LUAD, LUSC, PAAD, PRAD, READ, STAD, THCA, and UCEC (Fig 9B).

Subsequently, we employed a univariate Cox regression model to analyze the association between the expression levels of ACE and HSPB8 in pan-cancer samples and patient survival. We calculated the impact of each gene’s expression changes on patient survival risk and computed the corresponding HR and 95% CI. An HR value greater than 1 indicates that an increase in gene expression is associated with an increased risk of death for patients, suggesting the gene may act as a detrimental factor. Conversely, an HR value less than 1 suggests that an increase in gene expression is associated with a decreased risk of death, implying the gene may have a protective role.

Results show (Fig 9C and 9D) that the ACE gene has a significant detrimental effect in GBML, UVM, LGG, and LUSC samples and a significant protective effect in KIRC and SKCM. The HSPB8 gene has a significant detrimental effect in STAD, STES, UVM, COADREAD, BLCA, and BRCA and a significant protective effect in GBMLGG, SARC, ACC, LGG, KIRC, and LUAD.

### 3.9 HPA validation

**Fig 10:**
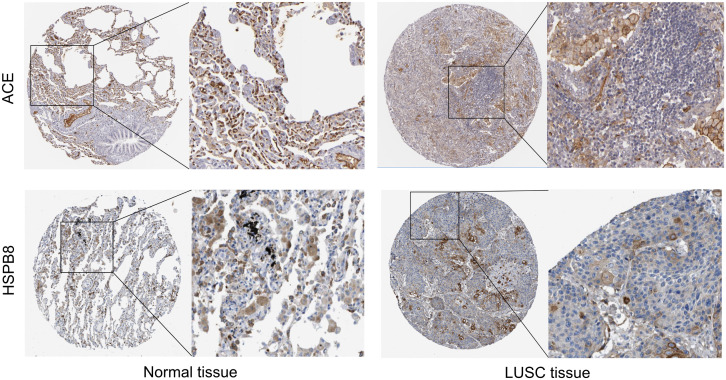
Validation of ACE and HSPB8 genes in the HPA database, the left-side shows normal tissues, and the right-side displays LUSC tissues.

Compared to normal tissues, both ACE and HSPB8 genes are significantly downregulated in LUSC, a finding further validated through the HPA database (Fig 10). This downregulation may play a crucial role in the onset and progression of LUSC.

### 3.10 Chemotherapy drug analysis

In this study, we analyzed the GDSC database to calculate the IC50 values of five commonly used chemotherapeutic drugs in LUSC treatment across different cancer cell lines. Our analysis revealed significant differences in the response to several key chemotherapeutic drugs between groups with varying levels of ACE gene expression.

Specifically, we observed significant differences in the IC50 values for cisplatin, paclitaxel, docetaxel, and vinorelbine between different risk groups (Figures A to E). For the HSPB8 gene, we also noted significant differences in drug sensitivity to cisplatin, gemcitabine, and vinorelbine (Figures F to J).

Notably, for both ACE and HSPB8 genes, lower IC50 values, indicating higher drug efficacy, were associated with patient groups exhibiting lower levels of gene expression. This finding underscores the potential value of these gene expression levels in predicting LUSC patients’ responses to specific chemotherapeutic agents.

**Fig 11:**
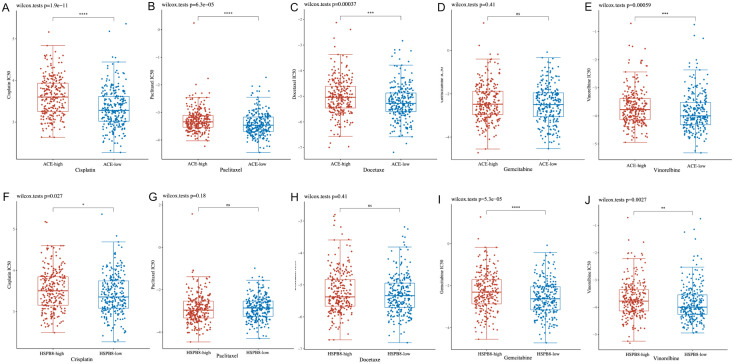
Distribution of IC50 scores for chemotherapeutic drugs in high and low-risk groups defined by ACE and HSPB8 gene expression, including cisplatin, paclitaxel, docetaxel, gemcitabine, and vinorelbine.

These results suggest that ACE and HSPB8 gene expression levels may serve as valuable biomarkers for predicting chemotherapy response in LUSC patients. The correlation between lower gene expression and increased drug sensitivity could have important implications for personalized treatment strategies in LUSC (Fig 11).

### 3.11 WB and cell experiments

**Fig 12:**
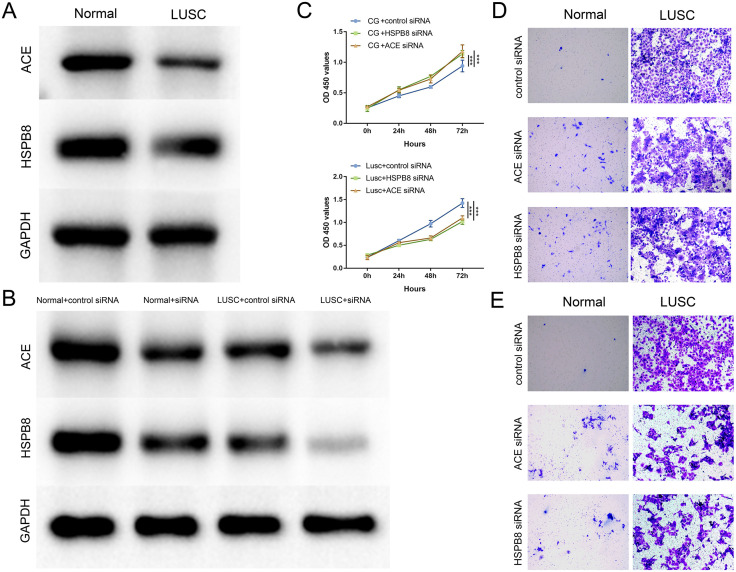
Validation of ACE and HSPB8 functions in normal tissue and LUSC. **(A)** WB analysis of ACE and HSPB8 expression levels in normal tissues and LUSC, with GAPDH serving as an internal control. **(B)** Validation of knockdown efficiency via WB experiments. **(C)** Time-course results of cell proliferation assessed by CCK-8 assay. **(D)** Results of cell migration assays. **(E)** Results of cell invasion assays.

This study aimed to elucidate the differential expression of specific proteins in the normal lung cell line BEAS-2B and the LUSC cell line H520, and their consequent impact on cellular behavior. Western blot analysis was initially used to determine the baseline expression levels of ACE and HSPB8 proteins in both normal and LUSC cells (Fig 12A). Subsequently, specific knockdown of these characteristic genes using siRNA technology was validated through Western Blot experiments, confirming knockdown efficiency (Fig 12B).

Six experimental groups were set up to observe distinct cellular behavior changes upon knockdown of ACE or HSPB8 in both normal and LUSC cells. The results were as follows:

1. Normal cells (BEAS-2B):

Post-knockdown, Matrigel-coated Transwell invasion assays demonstrated a significant enhancement in the normal cells’ ability to traverse the simulated basement membrane, indicating increased invasiveness. Similarly, significant increases in proliferation and migration behaviors were also exhibited by normal cells. These findings suggest that downregulation of ACE and HSPB8 in normal lung cells may promote cell growth and invasiveness (Fig 12C and 12D).

1. LUSC cells (H520):

Conversely, in LUSC cells, knockdown of ACE and HSPB8 resulted in a significant reduction in proliferation, migration, and invasion behaviors compared to the control siRNA treatment group (Fig 12C and 12E). This implies that these proteins may play roles in facilitating tumor growth and invasiveness in LUSC.

These contrasting results between normal and LUSC cells highlight the complex and context-dependent roles of ACE and HSPB8 in cellular behavior. In normal lung cells, these proteins appear to act as suppressors of proliferation, migration, and invasion. However, in LUSC cells, they seem to promote these behaviors, potentially contributing to tumor progression.

### 3.12 Somatic mutation landscape in HSPB8/ACE stratified subgroups

**Fig 13:**
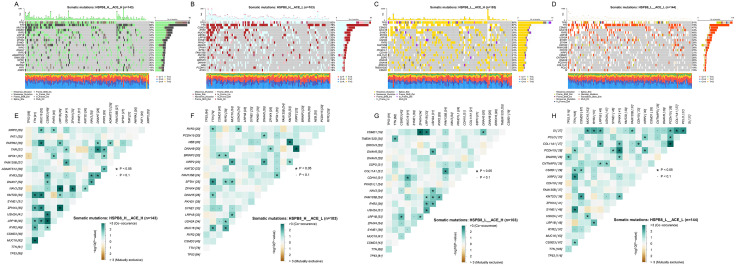
Somatic mutation landscape and co-occurrence analysis in HSPB8 and ACE expression-stratified samples. **(A–D)** Somatic mutation profiles of HSPB8_H_ACE_H, HSPB8_H_ACE_L, HSPB8_L_ACE_H, and HSPB8_L_ACE_L subgroups. **(E–H)** Co-occurrence and mutual exclusivity among the top 20 frequently mutated genes analyzed using Fisher’s exact test on 2 × 2 contingency tables, with odds ratios (ORs) quantifying effect direction and strength. P-values were adjusted for multiple comparisons using Benjamini–Hochberg FDR, with q < 0.05 considered significant. Co-occurring (positive) and mutually exclusive (negative) interactions are indicated by color, with symbols marking significant gene pairs.

To further investigate the genetic alterations underlying different HSPB8 and ACE expression subgroups, we analyzed the somatic mutation profiles across the four groups. As shown in Fig 13A–13D), distinct mutational landscapes were observed. The HSPB8_H_ACE_H subgroup (n = 143) exhibited frequent mutations in TP53, TTN, MUC16, CSMD3, and LRP1B. Similarly, the HSPB8_H_ACE_L group (n = 103) displayed mutations enriched in TP53, TTN, CSMD3, and RYR2. In contrast, the HSPB8_L_ACE_H group (n = 103) was characterized by alterations in TP53, TTN, MUC16, CSMD3, SYNE1, and RYR2, while the HSPB8_L_ACE_L subgroup (n = 144) showed recurrent mutations in TP53, TTN, MUC16, CSMD3, and RYR2.

We further assessed co-occurrence and mutual exclusivity among the top mutated genes (Fig 13E–13H). Significant co-occurring mutations were observed between TP53 and TTN, MUC16, CSMD3, or RYR2 in multiple subgroups (P < 0.05). In addition, subgroup-specific patterns were detected, such as the co-mutation of NAV3 and DNAH9 in the HSPB8_L_ACE_H group, and the co-occurrence of COL11A1 and CDH10 in the HSPB8_L_ACE_L group. These findings suggest that the mutation spectrum varies among HSPB8/ACE subgroups, potentially reflecting distinct molecular mechanisms and tumorigenic pathways.

## 4. Discussion

Recent studies have reported that high cumulative doses of ACE inhibitors (ACEIs) can significantly increase lung cancer incidence [49,50], with a higher risk observed among Asian patients. While the molecular mechanisms remain unclear, numerous publications have shown that ACE and ACE2 genes, part of the renin-angiotensin system (RAS), mediate cell proliferation, apoptosis, angiogenesis, and carcinogenesis [51,52]. The pulmonary vascular endothelium is the primary site for ACE-mediated metabolism of bioactive peptides. Lung cancer growth and metastasis can decrease pulmonary vessels, reducing ACE activity in lungs and serum [53], explaining the reduced ACE expression in lung cancer tissues. Although some studies have explored ACE gene polymorphism, no significant association with lung cancer has been found [54]. Given this complex landscape, we hypothesize that ACE’s role in lung cancer may be regulated by RNA expression levels rather than gene mutations, aligning with our mutation landscape analysis (S1 Fig). This hypothesis potentially explains the observed effects of ACEIs on lung cancer incidence and the reduced ACE expression in lung cancer tissues, while accounting for the lack of association between ACE gene polymorphisms and lung cancer.

The HSPB8 gene plays a critical role in various cellular processes. As a chaperone, HSPB8 aids in the correct folding of other proteins, especially under stress conditions, preventing the aggregation of misfolded proteins and mitigating cell toxicity [55]. HSPB8 has shown potential as a prognostic biomarker for cancer in numerous studies. It promotes gastric cancer cell growth by activating the ERK-CREB pathway, indicating poor prognosis [56]. In melanoma cells with BRAF and NRAS mutations, HSPB8 plays an anti-tumor role by regulating processes such as autophagy [57]. In LUAD, HSPB8 maintains mitochondrial function, promoting cell proliferation and migration [58]. Neither ACE nor HSPB8 has been systematically explored in carcinoma, underscoring the importance of further analyses and knockdown experiments in LUSC cells.

The TME in LUSC reveals a complex interplay between uncontrolled tumor cell proliferation and adverse environmental conditions, affecting ER functions in both cancer and infiltrating immune cells, resulting in ERS dysfunction [15]. Malignant cells experiencing ERS can coordinate immune evasion mechanisms, while tumor-infiltrating immune cells face restricted access to key nutrients [59]. The immune system’s tumor recognition and elimination involve coordinating metabolic, transcriptional, and translational programs in innate and adaptive immune cells. ER homeostasis regulation in tumor-infiltrating immune cells is critical for cancer progression and immune evasion [15]. Therapy-induced ERS can trigger ICD, stimulating anti-tumor immune responses [60]. Modulating key genes involved in ERS and UPR pathways may enhance immune checkpoint blockade and adoptive T cell immunotherapies in LUSC [16].

Despite evidence implicating the RAS and cellular stress responses in lung cancer and its microenvironment [61], the role of specific molecular regulators in LUSC remains insufficiently characterized. To narrow down potential drivers, we applied an intersection of feature selection and prognostic key gene screening. Through this approach, ACE and HSPB8 were identified for in-depth analysis through intersecting feature and prognostic key genes. ACE showed significant positive correlations with immune cells, suggesting potential in enhancing immune therapy efficacy. HSPB8 correlated positively with endothelial cells and macrophages, implicating its role in TME modulation. ACE’s negative correlation with TMB suggests its function in DNA integrity maintenance or damage repair. HSPB8 didn’t exhibit significant correlation with TMB, indicating distinct molecular pathways. Both ACE and HSPB8 showed significant negative correlations with mRNAsi, indicating involvement in tumor cell differentiation, growth rate, and invasiveness modulation, underscoring their potential as therapeutic targets in LUSC.

As previously mentioned, LUSC exhibits significant sample heterogeneity, which was highlighted in the pioneering study by Wu et al [62]. Compared to LUAD, another subtype of NSCLC, LUSC demonstrates notably higher internal heterogeneity characterized by changes in CNA, malignant cell distribution, and clonality. This heterogeneity poses a substantial challenge to the current classification systems and treatment paradigms, indicating an urgent need for a more detailed understanding of tumor biology, further evidenced by our co-clustering analysis. Complicating matters further, our research identified differential expression and functional roles of ACE and HSPB8 in both LUSC and LUAD. Notably, ACE appears to act as a detrimental factor in LUSC (p = 0.05, HR = 1.14, 95%CI (1.00, 1.30), potentially exacerbating tumor progression, whereas in LUAD, it tends to play a protective role (p = 0.39). Additionally, HSPB8 acts as a protective factor in LUAD (p = 0.05, HR = 0.89, 95%CI (0.80, 1.00) and tends towards a detrimental role in LUSC (p = 0.46). Therefore, the functions of ACE and HSPB8 in LUSC require further elucidation and validation.

The results of WB staining of cell lines for ACE and HSPB8 aligned with previous database findings. Subsequent knockdown experiments using siRNA for ACE and HSPB8, validated by WB, demonstrated that knocking down these genes in normal cell lines enhanced cancerous characteristics, whereas in LUSC cell lines, it inhibited cancerous properties. This dichotomy suggests a complex interplay of mechanisms. Primarily, ACE, associated with vascular proliferation in the circulatory system, and HSPB8, linked to mitochondrial function repair, may jointly regulate the cell cycle [63–65]. Knocking down ACE and HSPB8 affects cell proliferation by altering the expression or activity of cell cycle regulatory proteins. In normal cells, this intervention may promote excessive proliferation and transformation. In contrast, in LUSC cells, it could induce cell cycle arrest through impacting the Warburg effect, thereby inhibiting cell proliferation [66].

Interestingly, ACE and HSPB8 exhibit a paradoxical role: silencing promotes proliferative features in normal lung epithelial cells but inhibits malignant behaviors in LUSC cells. Such context-dependent dual functions are observed for other regulators, such as TGF-β, which suppresses early tumorigenesis but promotes invasion and metastasis in transformed cells [67]. Similarly, HSPB8 can act either as a tumor-promoting or tumor-suppressive factor depending on cancer type and mutational background [68,69], suggesting that differential pathway activation, feedback loops, and cell-type–specific metabolic or epigenetic states may underlie these contrasting effects. Tissue-specific regulation and embryonic origin may further contribute to the context-dependent roles of ACE and HSPB8 across cancers, warranting additional mechanistic investigation.

ACE and HSPB8 are interconnected: HSPB8 regulates autophagy and mitochondrial stress responses, while ACE modulates vasculogenesis. Autophagy and vascular remodeling are intertwined, exemplified by DCN-KDR/VEGFR2 interactions and AMPK-dependent pathways that inhibit VEGF-induced capillary morphogenesis [70,71]. These pathways may intersect with tumor metabolic reprogramming, as in the Warburg effect, which modulates macrophage ARG1 expression and suppresses T cell activation [72,73]. Although ER stress and epigenetic regulation initially guided gene selection, integrative model-based analyses identified ACE and HSPB8 as central to mitochondrial quality control, vascular regulation, and autophagy. Additionally, we initially considered including as many relevant genes as possible using both feature/prognostic intersections and literature-based strategies. Most literature-identified genes were also captured by GeneCards under our lenient threshold, so they were not annotated separately.

This study explored the multifaceted roles of ACE and HSPB8 in LUSC, revealing a dual-function paradox where their downregulation had opposing effects on normal versus cancerous cells. In normal cells, knockdown promoted proliferation and migration, while in LUSC cells, it inhibited these cancerous behaviors. This highlights the crucial role of tumor-specific context and the microenvironment in shaping therapeutic responses. Ongoing mechanistic investigations are profiling key signaling pathways, including MAPK/ERK and PI3K/AKT, assessing autophagy flux, and exploring epigenetic regulation across various LUSC cell lines to resolve these context-specific mechanisms. While this study’s primary focus was on developing a multi-gene scoring model and presenting novel findings, we acknowledge several limitations, including the reliance on a single survival database, potential signal averaging from batch effect correction, and a lack of external validation. Furthermore, the analysis was restricted to RNA expression without integrating genomic alterations, and mechanistic validation was limited to in vitro assays. Consequently, the moderate C-index and absence of in vivo validation mean that any claims regarding therapeutic applicability are preliminary and require further investigation. The ongoing experiments will extend these findings by elucidating pathway crosstalk and lineage-dependent regulation, providing a more comprehensive understanding to refine future therapeutic strategies. Future studies will incorporate exploratory approaches such as CRISPR-based screens to identify upstream regulators and downstream interactors of ACE and HSPB8, providing a roadmap for more comprehensive mechanistic studies and therapeutic development.

## 5. Conclusion

The ACE and HSPB8 genes emerge as promising diagnostic and prognostic molecular markers for LUSC. Yet, the delineation of their “comfort zones” through further exploration of molecular pathway functions and interventional experiments is imperative. Such studies are essential for refining personalized treatment strategies for cancer patients, offering valuable insights that could preliminarily enhance therapeutic outcomes.

## Supporting information

S1 Fig Landscape of genomic alterations in ACE and related RAS pathway genes across lung cancer cohorts. (TIFF)

S1 File Raw images. (PDF)
