# Supplementary figures and images for: Identification of ACE and HSPB8 as novel drug targets for LUSC treatment and prognosis based on a prognostic model integrating epigenetic regulation and endoplasmic reticulum stress-related genes
Source: PLoS One. 2026 Jan 5;21(1):e0335395. doi: 10.1371/journal.pone.0335395 (PMC12768259; doi:10.1371/journal.pone.0335395)

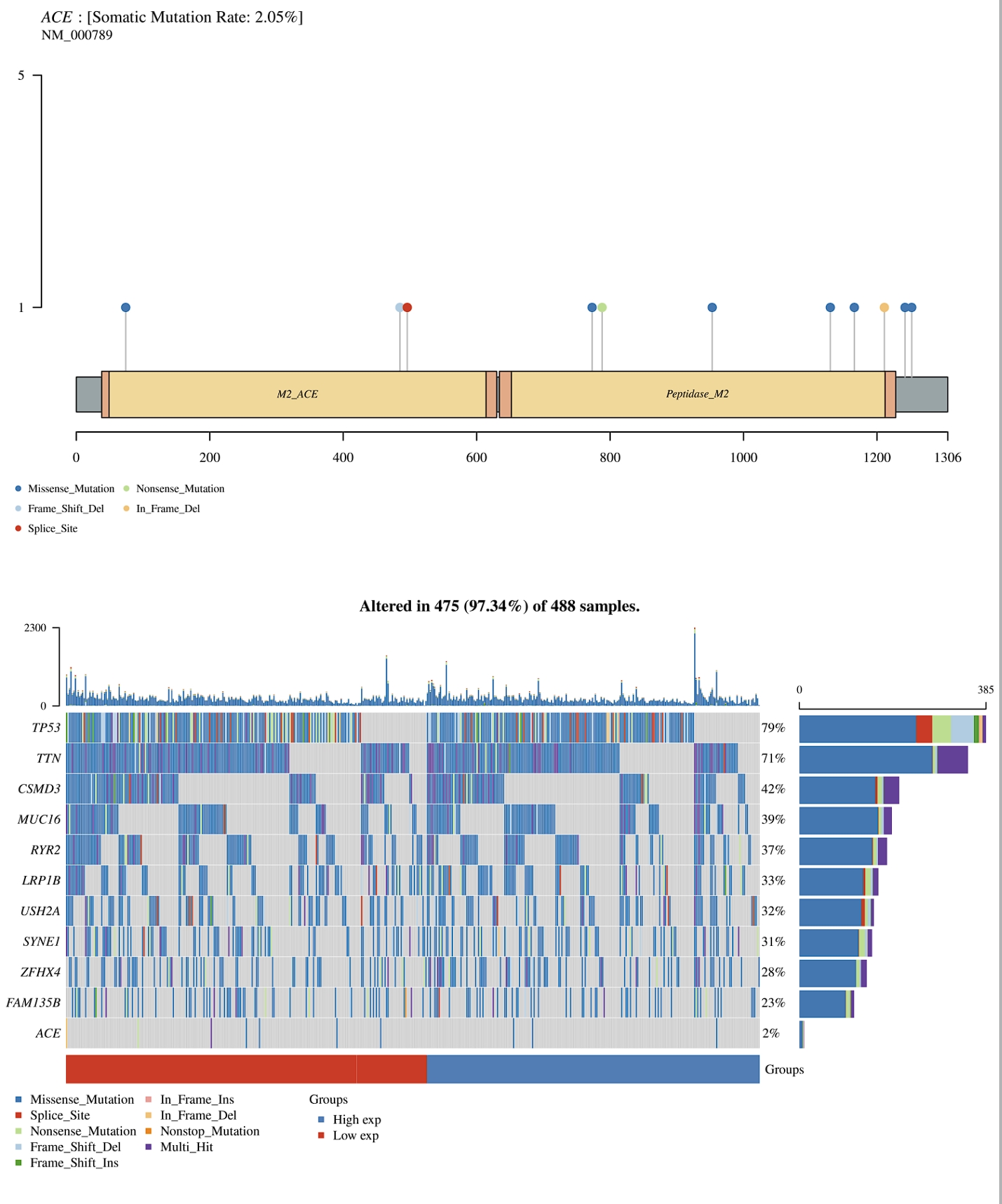

Supplement: S1 Fig — (TIFF) [file pone.0335395.s001.tiff]
