# Supplementary material for: Identification of ACE and HSPB8 as novel drug targets for LUSC treatment and prognosis based on a prognostic model integrating epigenetic regulation and endoplasmic reticulum stress-related genes
Source: PLoS One. 2026 Jan 5;21(1):e0335395. doi: 10.1371/journal.pone.0335395 (PMC12768259; doi:10.1371/journal.pone.0335395)
Supplement: S1 File — (PDF) [file pone.0335395.s002.pdf]

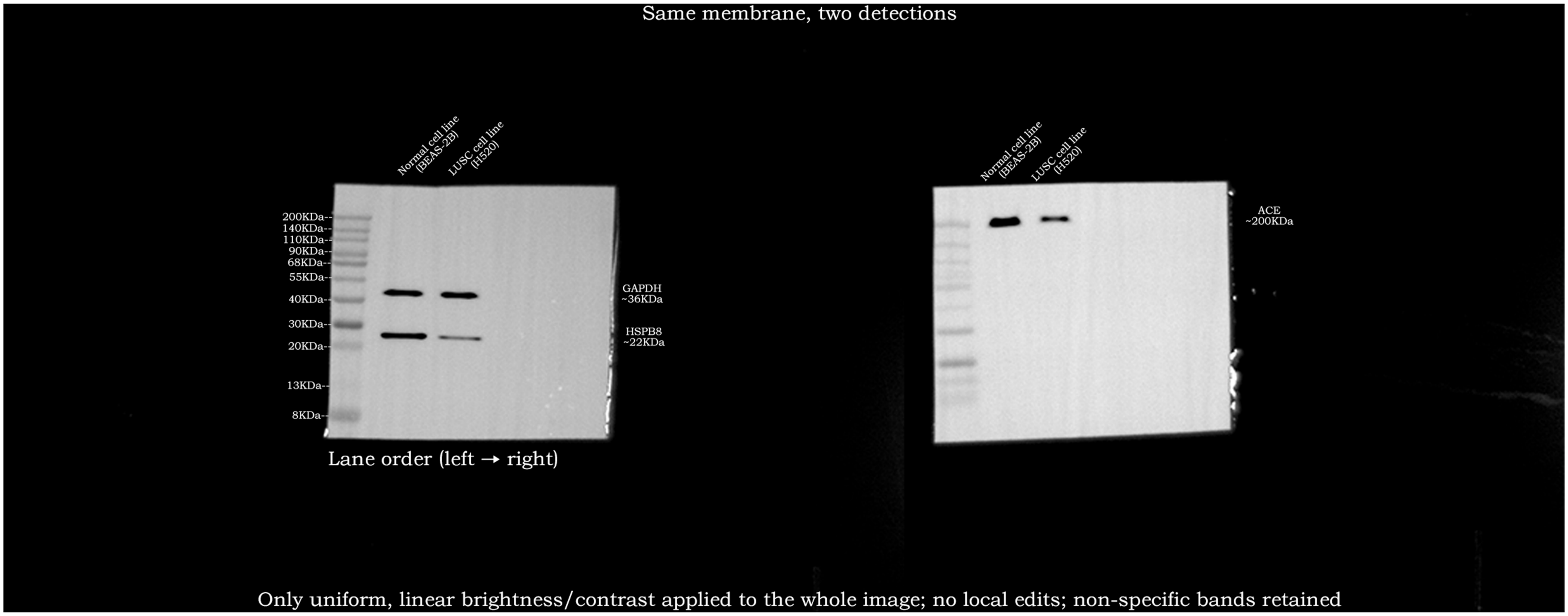

Same membrane, two detections
Normal cell line (BEAS-2B)
LUSC cell line (H520)
200KDa--
140KDa--
110KDa--
90KDa--
68KDa--
55KDa--
40KDa--
30KDa--
20KDa--
13KDa--
8KDa--
GAPDH ~36KDa
HSPB8 ~22KDa
Lane order (left → right)
Normal cell line (BEAS-2B)
LUSC cell line (H520)
ACE ~200KDa
Only uniform, linear brightness/contrast applied to the whole image; no local edits; non-specific bands retained

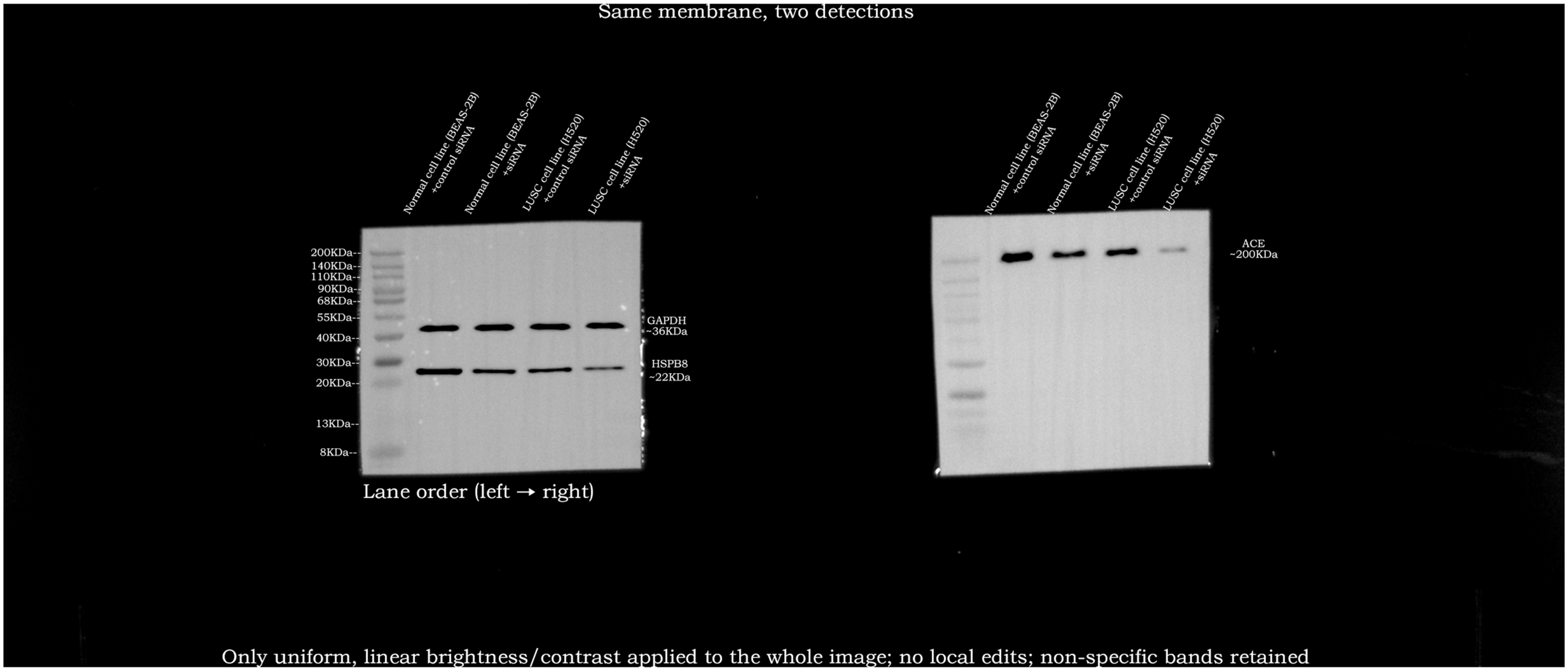

Same membrane, two detections
Normal cell line (BEAS-2B) +control siRNA
Normal cell line (BEAS-2B) +siRNA
LUSC cell line (H520) +control siRNA
LUSC cell line (H520) +siRNA
200KDa--
140KDa--
110KDa--
90KDa--
68KDa--
55KDa--
40KDa--
30KDa--
20KDa--
13KDa--
8KDa--
GAPDH ~36KDa
HSPB8 ~22KDa
Lane order (left → right)
Normal cell line (BEAS-2B) +control siRNA
Normal cell line (BEAS-2B) +siRNA
LUSC cell line (H520) +control siRNA
LUSC cell line (H520) +siRNA
ACE ~200KDa
Only uniform, linear brightness/contrast applied to the whole image; no local edits; non-specific bands retained
